# Pharmacodynamic mechanisms behind a refractory state in inflammatory bowel disease

**DOI:** 10.1186/s12876-022-02559-5

**Published:** 2022-11-17

**Authors:** Rasmus Goll, Øystein K. Moe, Kay-Martin Johnsen, Renate Meyer, Joachim Friestad, Mona D. Gundersen, Hege Kileng, Knut Johnsen, Jon R. Florholmen

**Affiliations:** 1 Research Group of Gastroenterology and Nutrition, Department of Clinical Medicine, UiT The Arctic University of Norway, Tromsø, Norway; 2 Department of Gastroenterology, Division of Internal Medicine, University Hospital of North Norway, Tromsø, Norway; 3 Department Internal Medicine, Vestre Viken Hospital, Hønefoss, Norway; 4 Department Internal Medicine, Hammerfest Hospital Hammerfest, Tromsø, Norway

**Keywords:** Anti-TNF therapy, Biological therapy, Crohn’s disease, Inflammatory bowel disease, Interleukin 17, Interleukin 23, Tumor necrosis factor, Ulcerative colitis, Ustekinumab, Vedolizumab

## Abstract

**Background and aims:**

Biological therapy for inflammatory bowel disease is efficient in many cases but not all. The underlying molecular mechanisms behind non-response to biological therapy in inflammatory bowel disease are poorly described. Therefore, we aimed to characterize the mucosal cytokine transcript profile in non-immunogenic, non-responder patients with adequate trough level.

**Material and methods:**

Patients with ulcerative colitis (UC) (*n =* 21) and Crohn’s disease (CD) (*n =* 12) with non-response to biological therapy (anti-tumor necrosis factor (TNF) or vedolizumab) were included. Reference groups were A: untreated patients with UC or CD at debut of disease who had severe 1-year outcome, B: patients with UC or CD treated to endoscopic remission with biological agents, and C: healthy normal controls. Mucosal transcripts of TNF, interleukin (IL)17 and IL23 were measured by reverse transcription real-time quantitative polymerase chain reaction. Results Of the non-responders, 2 out of 12 CD and 1 out of 21 UC patients needed surgery during follow-up. Of the remaining non-responding patients, 8 out of 10 CD and 12 out of 20 UC patients switched biologic treatment. The remaining 2 CD and 8 UC patients continued treatment with the same biological agent with the addition of steroids, immunomodulators (AZA/MTX) and /or local steroids/5ASA. Twelve (8 UC/4 CD) out of 20 IBD patients were still non-responders after changing biological therapy to either anti-TNF (2), vedolizumab (9) or ustekinumab (1).

The transcripts of IL17, IL23 and TNF were significantly upregulated in the non-response group compared to normal controls and patients in remission. In UC, 24% of the non-responders had normal mucosal TNF transcript indicating a non-TNF mediated inflammation. No obvious differences in gene expression were observed between primary and secondary non-responders, nor between anti-TNF and vedolizumab non-responders.

**Conclusions:**

Mucosal transcripts of IL17 and IL23 are highly associated with non-response to biological therapy, whereas some UC patients may also have a non-TNF mediated inflammatory pathway.

**Supplementary Information:**

The online version contains supplementary material available at 10.1186/s12876-022-02559-5.

## Introduction

Inflammatory bowel disease (IBD) includes the two entities ulcerative colitis (UC) and Crohn’s disease (CD). These are chronic inflammatory diseases believed to result from a dysregulated immune response caused by loss of immune tolerance in the gut. This is thought to be triggered by a combination of environmental and genetic factors, though the exact mechanisms are so far unknown [1]. Tumor necrosis factor (TNF) plays a central pathophysiological role in mediating the inflammation in IBD [2, 3]. This is underlined by the efficacy of anti-TNF therapy which is used in the most severe cases of both UC and CD [4, 5]. However, up to 30% of IBD patients do not respond to the initial anti-TNF treatment (primary non-response) whereas an additional 40% of the initial responders relapse during treatment (secondary non-response) [6, 7]. The failure of biologic therapy can either be due to well described pharmacokinetic mechanisms, such as inadequate tissue concentrations due to immunogenicity [8], or pharmacodynamic mechanisms that are poorly characterized in which the biologic agent is not targeting the activated inflammatory pathway.

Except for immunogenic treatment failure, we have little knowledge of how the IBD inflammasome can escape the apoptotic and immunosuppressive effects of anti-TNF therapy. Recently, attention has been brought to the IL23/IL17 pathway that could represent heterogeneity in disease pathobiology and thus explain the efficacy of anti-IL23 in the treatment of some anti-TNF refractory patients with CD [9].

Therefore, there is an apparent lack of our understanding of the molecular mechanisms behind the pharmacodynamically related refractory state in patients treated with biological therapy [10]. In this study we aimed to characterize the clinical features of non-responders in our clinical centers and investigate the mucosal gene activations of TNF, IL17 and IL23 in UC and CD patients refractory to biological therapy.

## Materials and methods

### Patients and biopsy sampling

Patients aged ≥18 years were recruited in the time period 2015–2020 from 3 clinical centers in Norway (Gastrointestinal units at the hospitals of Hammerfest, University Hospital North Norway in Tromsø, and Ringerike, Hønefoss as a part of an ongoing prospective study - Advanced Study of Inflammatory Bowel disease (ASIB- study).

The participants signed an informed consent. All methods were performed according to the Helsinki declaration. Approval including the use of biobank was granted by the Regional Committee of Medical Ethics of Northern Norway Ref no: 1349/2012.

### Reference groups

Five reference groups were included from the ASIB biobank. **1.** 13 UC patients in remission: UC patients treated to remission by biologic agents with Mayo endoscopic score 0–1 and normalized mucosal TNF transcript; **2.** 15 CD patients in remission: CD patients treated to remission by biologic agents with SES-CD-score 0,CDAI < 150 and normalized mucosal TNF transcript; **3.** 13 untreated UC patients at debut of disease who had a severe outcome (biologics or surgery within first 12 months after debut); **4.** 10 untreated CD patients (like group **3** but with CD); and **5.** 15 healthy controls: subjects performing a cancer screening examination with no clinical, endoscopic or histological signs of intestinal disease. For groups **3** and **4**, samples for cytokine transcript analysis were taken at debut of disease before any treatment was initiated.

### Diagnosis and clinical grading of activity

The diagnosis of UC and CD was based on established clinical, endoscopic and histological criteria [11]. The degree of illness was evaluated using the clinical scoring system ulcerative colitis activity index (UCDAI) [12], Crohn’s Disease Activity Index (CDAI) [13], Simple endoscopic score in CD (SES-CD) [14] and the Montreal classification of IBD [15].

### Criteria for non-response to biological therapy

The criteria for non-response (both primary and secondary) to biological therapy were: adequate duration of treatment, serum concentrations of biologic agents at therapeutic levels, no pathogenic bacteria detected in fecal samples and active disease according to both endoscopic (inflammation and/or ulcer) and clinical (UCDAI-score > 2 or CDAI-score > 150) assessment. Biopsies were taken before changing treatment.

Adequate treatment duration for anti-TNFs was set at a minimum of 8 weeks with infliximab (IFX) and 12 weeks with adalimumab (ADA) due to the possibility of successful induction after treatment with infliximab-infusions weeks 0,2 and 6 or after 3–5 bi-weekly adalimumab -injections [16]. To be noted, in the induction phase patients treated with ADA received either 160 mg and 80 mg s.c. in the first and second week,respectively, or 80 mg s.c. weekly the first three weeks, thereafter bi-weekly 40 mg injections. For golimumab (GOL) a minimum of 14 weeks of treatment was required prior to inclusion [17]. Studies have shown that more than 50% of reported patients treated with these anti-TNFs (IFX, ADA, GOL) achieved response within 6–8 weeks of treatment [16].

As for ustekinumab (UST), treatment for at least 16 weeks was required [18]. Regarding vedoluzimab (VDZ), an anti-α4β7 integrin antibody, the required duration of treatment was based on the findings in the GEMINI (I-III)- studies [19] and others [20]. Thus, treatment for a minimum of 14 weeks qualified for inclusion.

### Treatment of non-response to biological therapy

The non-responders to biological therapy were evaluated by their treating physician to either be in need of surgery, switch of biologic agent or addition of systemic steroids, immunomodulators (AZA/MTX) or local 5-ASA/corticosteroids. Switching of biologic agent entailed either treatment with a drug within the same class (eg. another anti-TNF) or a change of mechanism to either anti-integrin or anti-IL12/23.

Treatment responses in UC were defined as follows; Remission: An UCDAI score < 3 including an endoscopic subscore of 0–1. Response: An UCDAI improvement of at least 3 [21].

Treatment responses in CD were defined as follows; Remission: No endoscopic ulcers and no redness with CDAI score < 150 [8]. Response: A CDAI score improvement of at least 70 [13].

### Tissue samples

Colonic mucosal biopsies were sampled with standard forceps from either the region with the most severe inflammation (non-responders and UC/CD patients with severe outcome after 1 year) or from normal mucosa (healthy controls and patients in remission). For patients in remission the samples were taken from the area with previously most severe inflammation. Biopsy specimens for RNA extraction were immediately immersed in RNA *later* (Qiagen) and stored at room temperature overnight, then at − 20 °C until RNA isolation.

Total RNA was isolated from patient biopsies using the Allprep DNA/RNA Mini Kit (Qiagen, Hilden, Germany, Cat No: 80204) and the automated QIAcube instrument (Qiagen, Hilden, Germany) according to the manufacturer’s recommendations. Quantity and purity of the extracted RNA were determined using the Qubit 3 Fluorometer (Cat No: Q33216; Invitrogen by Thermo Fisher Scientific, Waltham, MA, USA). Reverse transcription of the total RNA was performed using the QuantiTect Reverse Transcription Kit (Cat. No: 205314; Qiagen, Hilden, Germany). Real-time PCR procedures have previously been described in detail [22]. Beta-actin (ACTB) was used as housekeeping gene. The fold-difference of IL17 and IL23 expressions were calculated according to the ΔΔCT-method. Cross-plate adjustment was done using an interplate calibrator. TNF absolute quantification was analysed using an already established in-house method [23]. The following cytokine transcripts were measured: TNF, IL17, and IL23 (primer sequences previously published [22]).

### Statistics

All calculations were performed in IBM SPSS Statistics for Windows, Version 28.0. Armonk, NY: IBM Corp. Values not following a Gaussian distribution were logarithmically transformed before testing group differences. Group differences were tested by two-way ANOVA with adjustment for sex and age, and post-hoc adjustment for multiple comparisons ad modem Sidak. We entered delta-CT values (delta-CT = target gene CT – ACTB CT) for IL17 and IL23, and logarithmically transformed copy number for TNF-values. To be noted, the TNF-values of patients in remission were omitted from statistical analysis as the TNF values was used as selection criterion for these patient groups. TNF values were adjusted for ACTB expression on CT level before applying an absolute standard curve for calculating copy number. *P*-values < 0.05 were considered significant.

## Results

### Clinical descriptors

The demographics of the included 47 UC patients, 37 CD patients and 16 healthy controls are shown in Table 1.

**Table 1:** Characteristics of ulcerative colitis (UC) and Crohn’s disease (CD) patients with non-response to biological treatment, severe outcome, remission and normal controls. Values are in actual number or mean (range). For further details, see text. * The Montreal classification for IBD. # Mucosal TNF expression below 7500 copies/μg total RNA was a selection criterium for these groups

Patient groups	UC non-response	CD non-response	UC severe outcome	CD severe outcome	UC remission	CD remission	Normal controls
Number	*n =* 21	*n =* 12	*n =* 13	*n =* 10	*n =* 13	*n =* 15	*n =* 16
Age	40.3 (18–71)	43.1 (27–56	34.4 (22–67)	37.2 (22–58)	48.4 (20–76)	36.7 (20–54)	52.8 (19–83)
Sex (female/male)	7/14	4/8	7/6	5/5	4/9	8/7	7/9
Smoking current/earlier/never	2/5/7	2/7/1	0/5/6	2/2/4	1/6/3	1/1/5	3/7/4
Area involved (UC) E1/E2/E3*	1/9/11		2/6/5		0/7/6		
Area involved (CD) L1/L2/L3/L4*		1/4/7/0		2/3/4/1		4/5/6/0	
Duration of disease (years)	8.7 (1–35)	11.5 (3–40)			8.8 (2–28)	7.9 (2–24)	
Treatment duration (weeks)	91.4 (17–270)	154.3 (21–634)			149 (47–432)	133 (50–321)	
Anti-TNF/VDZ/UST	21/0/0	10/2/0			13/0/0	13/1/1	
Primary/secondary non-response	12/9	10/2					
Mucosal TNF gene transcription	17,923 (1700–61,100)	21,692 (7600–46,400)	19,100 (12600–30,700)	27,970 (4000–63,400)	#4088 (350–7000)	#3607 (200–6100)	4869 (300–11,400)
UCDAI/CDAI score	9.4 (3–12, *n =* 19)	279 (180–483, *n =* 10)	9.8 (6–12)	239 (176–295, *n =* 4)	0,3 (0–1, *n =* 11)	37 (0–114, *n =* 8)	
MAYO/SES score	2.7 (2–3)	10.9 (3–22)	2.5 (2–3, *n =* 11)	10 (9–11, *n =* 2)	0,3 (0–1, *n =* 11)	0 (0)	
Calprotectin mg/kg	1030 (50–3000)	821 (60–3000)	1366 (240–3000)	648 (25–1585)	46 (25–150, *n =* 11)	48 (20–185, *n =* 13)	25 (< 25–25, *n =* 3))
Fecal bacterial culture performed (yes/no)	11/10	4/8					
Concentration of biologic agent measured (yes/no)	19/2	10/2					

#### Ulcerative colitis non-response

21 patients were included (7 female/ 14 male, average age 40,3 years, range 18–71 years), 12 patients with primary and 9 patients with secondary non-response to IFX (9 patients), ADA [9], GOL [3]. One patient who was included as a non-responder to GOL, did not respond to VDZ either. One of the UC patients who needed a colectomy swithched from IFX to ADA prior to the operation, but received treatment with ADA for an insufficient amount of time (8 weeks) to be classified as a non-responder to retreatment. In total 21 UC non-responders were eligible for study inclusion and cytokine analyses.

#### Crohn’s disease non-response

12 patients with CD were included (4 female/8 male, average age 43,1 years, range 27–56 years), 10 patients with primary and 2 patients with secondary non-response to IFX (5), ADA (1), GOL (4) and VDZ (2). One patient who was included with non-response to GOL did not respond to ADA nor UST. One CD patient achieved response when switching from IFX to ADA. In total 12 CD non-responders were entered in the cytokine analyses.

### Further treatment and clinical outcome

**Fig. 1:**
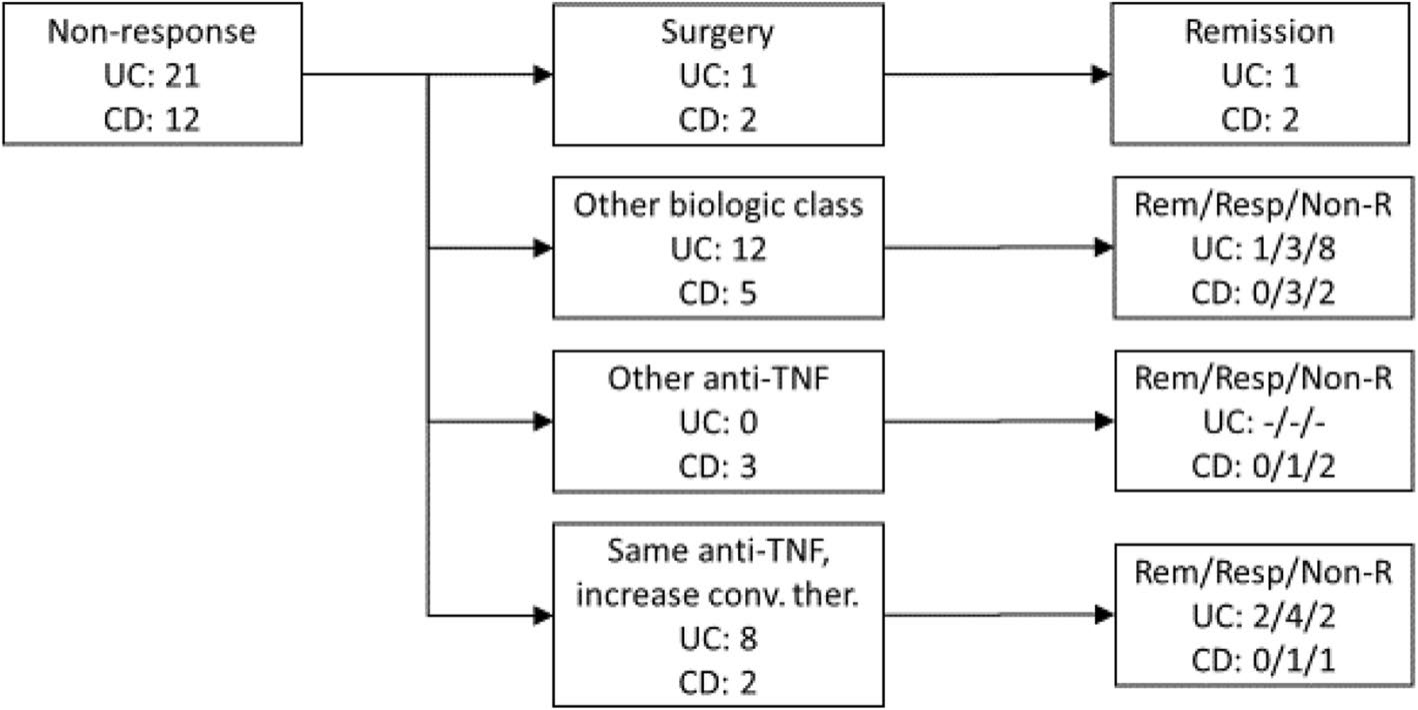
Clinical outcome of ulcerative colitis (UC) and Crohn’s disease (CD) with non-response to biological treatments. Conventional therapy includes 5-ASA local/systemic, corticosteroid local/systemic, immunomodulator (azathioprine or methotrexate)

In Fig. 1 further treatment and the clinical outcomes of the non-responders are shown. Of the UC and CD patients with non-response 1 out 21 (5%) and 2 out of 12 (17%) were in need of surgery, respectively. The remaining patients were either switched to a different biologic agent or given concomitant steroids, immunomodulators and/or 5ASA. Of those who received treatment with vedoluzimab or ustekinumab 10 out of 17 (59%) were still non-responders. Eight patients with UC retained their current biologic treatment with an increase in conventional therapy (systemic steroids, immunomodulators, 5ASA and/or local steroids/5ASA). Of these 2 achieved remission and 4 obtained response. Two patients with CD retained their current biologic treatment with addition of systemic steroids or immunomodulators. Of these 1 obtained response.

### Mucosal cytokines gene transcription

For the cytokine transcript analysis only samples from the first non-responder event was used.

#### Ulcerative colitis

**Fig. 2:**
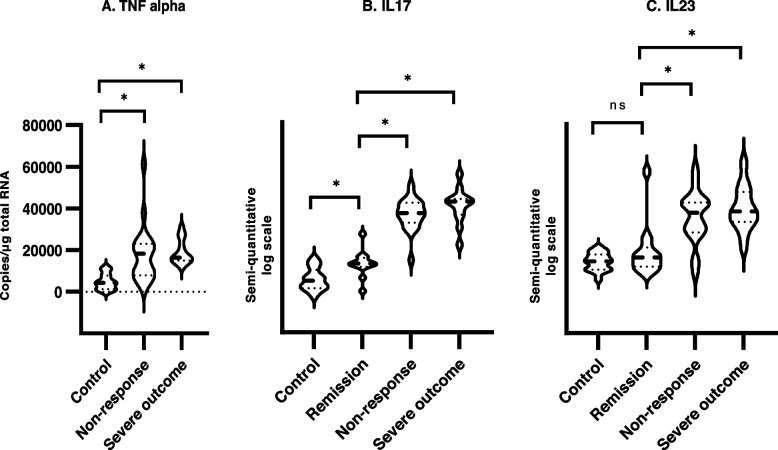
Colon mucosa cytokine gene transcripts of TNF, IL17 and IL23 in normal controls and patients with ulcerative colitis in remission, with non-response to biologic treatment, and treatment naïve at debut who had a severe 1-year outcome. TNF is given in copies/μg total RNA, while IL17 and IL23 is presented on a semi-quantitative log scale (inverse ΔCT). Difference between groups were performed by two-way ANOVA adjusting for age and sex, posthoc comparisons with Sidak adjustment. Ulcerative colitis

The mucosal TNF transcript was 4.0 [95% confidence interval: 1.9–8.7] times higher in the non-responder group compared to the healthy control group (*p <* 0.001). No difference was observed when comparing the group of UC at debut with severe outcome with the non-responder group (*p =* 0.54) (Fig. 2A). Of note, 5 of the non-responder patients (2 extensive, 3 left sided) had normal mucosal TNF transcript levels. In comparison, patients at debut of disease with severe 1-year outcome had 5.9 [2.5–14.3] times higher TNF expression than healthy controls (*p <* 0.001).

The mucosal transcripts of IL17 were 67.9 [24.8–186.2] times higher in the non-responder group than in the healthy control group (*p <* 0.001), and 20.1 [7.8–56.1] times higher than the remission group (*p <* 0.001), whereas no differences were observed compared to the group of UC patients with severe outcome (*p =* 0.586) (Fig. 2B). Interestingly, patients in remission had 3.2 [1,1–9.8] times higher IL17 expression than healthy controls (*p =* 0.032).

Transcripts of IL23 parallelled the IL17 differences at lower levels. The non-responder group had IL23 transcripts 6.0 [2.6–13.7] times higher than healthy controls (*p <* 0.001), and 3.9 [1.6–9,5] times higher compared to patients in remission (*p <* 0.001)(Fig. 2C). Patients at debut of disease with severe 1-year outcome had 9.7 [3.8–24.8] times higher IL23 expression compared to controls (*p <* 0.001).

No obvious cytokine transcript differences were observed between primary and secondary non-responders, nor between anti-TNF and VDZ non-responders.

In the abovementioned subgroup of UC patients with normal mucosal TNF transcripts, 2 switched to VDZ without response and 3 continued with antiTNFs with the addition of steroids/immunomudulators or 5ASA. Of these, 1 achieved remission and 2 obtained response.

The transcripts of IL17 were higher in UC patients with non-response and severe outcome compared to the corresponding CD patient groups (*p =* 0.029 and *p* > 0.001, respectively). Regarding the transcripts of IL23, these were increased in the group of UC patients with severe outcome and in remission as compared to the corresponding CD patient groups (*p =* 0.001 and *p =* 0.02, respectively). There were no significant differences in the transcripts of TNF between the groups of UC and CD patients.

#### Crohn’s disease

**Fig. 3:**
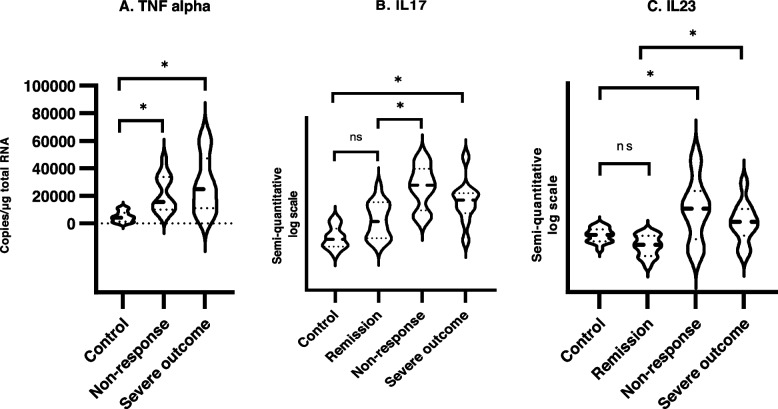
Intestinal mucosa cytokine gene transcripts of TNF, IL17 and IL23 in normal controls and patients with Crohn’s disease in remission, with non-response to biologic treatment and treatment naïve at debut who had a severe 1-year outcome. TNF is given in copies/μg total RNA, while IL17 and IL23 is presented on a semi-quantitative log scale (inverse ΔCT). Difference between groups were performed by two-way ANOVA adjusting for age and sex, posthoc comparisons with Sidak adjustment. Crohn’s disease

The mucosal TNF transcript was 5.0 [1.8–14.0] times higher in the non-responder group than in the group of healthy controls (*p =* 0.001), but the level was not different from that of CD at debut with severe outcome (*p =* 0.967)(Fig. 3A). None of the patients with non-response had normal mucosal transcript levels of TNF. Patients at debut of disease with severe 1-year outcome had 6.0 [1.9–18.5] times higher TNF transcript levels compared to healthy controls (*p =* 0.001).

The mucosal IL17 transcripts were 29.9 [6.6–134.9] times higher in the non-responder group compared to the healthy controls (*p <* 0.001), and 7.3 [1.8–29.1] times higher than patients in remission (*p =* 0.002)(Fig. 3B). The level of IL17 transcript was 14.4 [2.9–72.0] times higher in the group of CD patients with severe outcome compared to healthy controls (*p <* 0.001), but not compared to CD patients in remission (*p =* 0.089).

The mucosal IL23 transcript levels in the non-responders were 3.1 [1.2–8.3] times higher than in the healthy control group (*p =* 0.014), and 4.2 [1.6–11.2] times higher than patients in remission (*p =* 0.001) (Fig. 3C). Furthermore, the mucosal transcript of IL23 was 3.4 [1.3–9.0] times higher in CD patients with severe outcome compared to patients in remission (*p =* 0.007), but not compared to healthy controls (*p =* 0.112).

There were no significant differences in the levels of IL17 and IL23 between CD patients with non-response and patients with severe outcome .

No obvious cytokine differences were observed between primary and secondary non-responders, nor between anti-TNF and VDZ non-responders.

## Discussion

In this study of IBD patients, pharmacodynamic mechanisms appear more likely responsible for the non-response to biological therapies including anti-TNF and vedolizumab. All patients were non-immunogenic and had adequate trough levels of given therapy.

Two main findings were observed in the cytokine analysis. Firstly, the mucosal transcripts of TNF, IL17 and IL23 transcripts were significantly increased in IBD patients with active inflammation regardless of treatment. These findings are in consistence with their role as mainly proinflammatory cytokines. Pre-treatment transcripts of IL17, IL23 and TNF from the same patients would possibly yielded further insights into the mechanisms behind a treatment-refractory state. Alas, these were not obtained. Nevertheless, we demonstrated an association between non-response to biologic agents and an increased transcription of these cytokines. This is as expected, but could indicate possible resistance mechanisms to biological therapy.

Secondly, in the UC group, unlike the CD group, 24% had normal mucosal TNF transcript suggestive of a non-TNF mediated inflammation. These observations imply a potential for patient stratification and more precise therapeutic strategies.

It is well documented that IL-23 with subsequent activation of IL-17 plays a pivotal role in mediating the inflammation in IBD [24]. Moreover, we have previously shown that IL23/IL17 transcript levels correlate well to the grade of inflammation in IBD [2] and treatment with anti-TNF to complete endoscopic remission has shown to effectively reduce these cytokines in CD [25] and in UC [22]. For UC patients in remission the mucosal transcript of IL17 was slightly increased compared to controls while no significant differences were detected between these groups regarding IL23. In CD patients in remission there was a tendency of lower IL23 transcripts versus controls.

Between UC and CD patients there were tendencies towards higher transcripts of IL17 and IL23 in the UC groups, but not regarding the transcript of TNF. Further subanalysis between patients treated with other biologics than anti -TNFs (ie. non-responders and patients in remission) was not possible due to the low number of observations.

IL 17 is a cytokine with complex functions involved in both inflammation as well as gut epithelial cell integrity [26]. Consequently, this increased mucosal transcript of IL 17 could either represent a persisting low-degree inflammation or be attributed to the epithelial healing process. Thus, the mucosa of patients in remission seem to differ slightly from the mucosa of controls. This could be of clinical significance, particularly regarding time to relapse.

The mechanism of action for anti-TNF is poorly understood. Evidence suggests that anti-TNF binds to immune cells expressing membrane-bound TNF (mTNF) [27, 28]. Already in 2003 the anti-TNF infliximab was shown to induce T-cell apoptosis in lamina propria [29], possibly via TNF receptor 2 and intestinal CD14+ macrophages [30].

The mTNF/TNFR2 signalling pathway has been further investigated by Schmitt et al. [31]. In their study apoptosis-resistant intestinal TNFR2 + IL23R+ T cells were associated with resistance to anti-TNF therapy in Crohn’s disease. Moreover, IL23 appears to promote apoptosis-resistant T cells and drug resistance, at least in CD (for review, see [24]). Therefore, this may be one of several explanations for the apparent escape from the immunosuppressive effects observed in the non-responders to biological therapy. An observation supporting this hypothesis is the report by Cravo et al. where IL23R polymorphisms influence phenotype and response to therapy in UC [32].

We cannot fully exclude that non-response could be explained by too low mucosal concentration of the drug. Leakage of active substance (antibodies) may be a mechanism which could explain discrepancy between adequate serum levels of biologic agents and non-response [33]. High mucosal levels of TNF transcripts despite anti-TNF therapy can also indicate the need to reevaluate therapeutic levels of anti-TNF’s. In our department we perform an algorithm of intensified induction treatment until remission is achieved which gives high concentrations in the blood [34]. Loss of active substance into the lumen may counteract this strategy. Measuring the mucosal level of the biologic agent in patients with non-response could be of value, especially when the concentration in the blood is high.

The other main finding in our study was a normalized mucosal TNF transcript despite endoscopic inflamed mucosa in 24% of the UC non-responders to anti-TNF. Of these, only those who continued anti-TNF with the addition of steroids/immunomodulators and/or 5-ASA achieved response/remission. This implies that non-TNF mediated inflammatory mechanisms can be seen in IBD, also described as *non-TNF driven disease* [35]. None of the non-responder CD patients had normal mucosal TNF transcript.

The strength of this study is that we have characterized both the clinical aspects of non- immunogenic non-responders to biological therapy and their respective mucosal immunologic phenotype with cytokines of most interest in regards to potential immunological escape. The most obvious weaknesses are the low number of patients and the lack of a deeper mechanistic approach to describe both the IL23-R mechanisms and its relation to the cases with normal mucosal TNF transcripts. This awaits further studies.

## Conclusion

Non-response to biological therapy in IBD independent of immunogenicity appears both to anti-TNF, vedolizumab and ustekinumab. Mucosal transcripts of IL17 and IL23 are highly associated with non-response to biological therapy, whereas some patients may also have a non-TNF mediated inflammatory pathway.

## Supplementary Information

**Additional file 1.**

## Data Availability

The raw data are supplied in a supplementary file.

## Abbreviations

ASIB: Study, Advanced Study of Inflammatory Bowel disease
TNF: Tumor necrosis factor
UCDAI: Ulcerative colitis activity index
CDAI: Crohn’s disease activity score
AZA: Azathioprine
MTX: Methotrexate
5ASA: 5: Aminosalicylic acid
